# Reptilian Transcriptomes v2.0: An Extensive Resource for Sauropsida Genomics and Transcriptomics

**DOI:** 10.1093/gbe/evv106

**Published:** 2015-07-01

**Authors:** Athanasia C. Tzika, Asier Ullate-Agote, Djordje Grbic, Michel C. Milinkovitch

**Affiliations:** ^1^Laboratory of Artificial & Natural Evolution (LANE), Department of Genetics & Evolution, University of Geneva, Switzerland; ^2^SIB Swiss Institute of Bioinformatics, Switzerland; ^3^Institute of Genetics and Genomics of Geneva (iGE3), University of Geneva, Switzerland

**Keywords:** reptiles, transcriptomes, deep sequencing, deep sequencing, squamates, turtles, turtles, Archosauria

## Abstract

Despite the availability of deep-sequencing techniques, genomic and transcriptomic data remain unevenly distributed across phylogenetic groups. For example, reptiles are poorly represented in sequence databases, hindering functional evolutionary and developmental studies in these lineages substantially more diverse than mammals. In addition, different studies use different assembly and annotation protocols, inhibiting meaningful comparisons. Here, we present the “Reptilian Transcriptomes Database 2.0,” which provides extensive annotation of transcriptomes and genomes from species covering the major reptilian lineages. To this end, we sequenced normalized complementary DNA libraries of multiple adult tissues and various embryonic stages of the leopard gecko and the corn snake and gathered published reptilian sequence data sets from representatives of the four extant orders of reptiles: Squamata (snakes and lizards), the tuatara, crocodiles, and turtles. The LANE runner 2.0 software was implemented to annotate all assemblies within a single integrated pipeline. We show that this approach increases the annotation completeness of the assembled transcriptomes/genomes. We then built large concatenated protein alignments of single-copy genes and inferred phylogenetic trees that support the positions of turtles and the tuatara as sister groups of Archosauria and Squamata, respectively. The Reptilian Transcriptomes Database 2.0 resource will be updated to include selected new data sets as they become available, thus making it a reference for differential expression studies, comparative genomics and transcriptomics, linkage mapping, molecular ecology, and phylogenomic analyses involving reptiles. The database is available at www.reptilian-transcriptomes.org and can be enquired using a wwwblast server installed at the University of Geneva.

## Introduction

The fields of genomics and transcriptomics maintain their fast development thanks to the improvement of sequencing technologies and associated bioinformatic tools for assembly and annotation. The availability of deep-sequencing techniques that produce hundreds of thousands to millions of reads of variable sizes, at only a fraction of the time and cost of Sanger sequencing, is leading to the accumulation of data in an increasing number of species. Despite these technological advances, sequencing data remain unevenly distributed across phylogenetic groups; for example, 50 of the 72 vertebrate genomes currently available in Ensembl belong to mammals (Flicek et al. 2014), most of them originating from the “Mammals Genome Project” (Lindblad-Toh et al. 2011), even though mammals comprise only 8% of the total number of vertebrate species. Current sequencing initiatives aim at a better coverage of eukaryotic lineages with, for example, the “Genome 10k Project,” the “i5k initiative,” and the “959 Nematode Genomes” proposing to sequence the genomes of 10,000 vertebrate, 5,000 insects and 959 nematode species, respectively (Genome 10K Community of Scientists 2009; Kumar et al. 2012; i5K Consortium 2013).

Although the availability of genome sequence data in a broad range of species would greatly facilitate functional evolutionary and developmental studies, these proposals will require a considerable amount of time to yield high-quality genomic resources. To overcome the high cost and difficulties of de novo genome sequencing and annotation, analyzing transcriptomes is an appealing alternative: Less sequencing data and computational resources are required. To generate a reference data set of annotated genes of a nonmodel species, one can pool RNA from multiple tissues, developmental stages and individuals for the preparation of complementary DNA (cDNA) libraries (Ekblom and Galindo 2011), thus sequencing a maximum number of genes. Special attention should be given to the selection of tissues for mRNA extraction to ensure that a high number of transcripts is expressed and sequenced (Ramskold et al. 2009). The transcriptome can be further enriched by the normalization of the cDNA libraries, as it equalizes the abundance of expressed transcripts by decreasing the proportion of the most highly expressed mRNAs, hence, facilitating the identification of poorly transcribed genes (Zhulidov et al. 2004).

Several freely available and efficient de novo transcriptome assemblers are available (e.g., Trans-Abyss [Robertson et al. 2010], Oases [Schulz et al. 2012], and Trinity [Grabherr et al. 2011]) that take into account the particularities of transcriptome sequence data sets, such as the variation in sequencing depth due to the variable abundance of transcripts and the presence of multiple transcript variants because of alternative splicing (Martin and Wang 2011). Annotation of the assembled transcripts is probably the most difficult step. Indeed, as functional analyses of transcripts are rarely undertaken in a systematic fashion, the identification of genes relies heavily on homology inference based on similarity analyses against already characterized sequences from other species. Even though the most widely used program for this task is the Basic Local Alignment Search Tool (BLAST; Altschul et al. 1990), a variety of protocols (type of BLAST, search parameters, databases inquired) are used in published studies, thus hindering the comparison of results. Furthermore, the sequence data provided with a specific publication vary extensively from none to raw deep-sequencing reads to annotated full transcripts. The only current database that integrates large-scale comparative genomic analyses within a single analytical and phylogenetic framework is Ensembl (Kersey et al. 2014). Yet, only high-quality fully sequenced genomes are incorporated in Ensembl, creating the necessity to generate smaller databases that target insufficiently represented lineages.

Among the most underrepresented vertebrate groups in terms of genomic data is the paraphyletic Class Reptilia. With more than 10,000 described species (http://www.reptile-database.org, last accessed June 10, 2015), only 12 genomes have been fully sequenced with varying quality: The Anole lizard *Anolis carolinensis* (Alfoldi et al. 2011), the Chinese softshell turtle *Pelodiscus sinensis* and the Green sea turtle *Chelonia midas* (Wang et al. 2013), the Western painted turtle *Chrysemys picta* (Shaffer et al. 2013)*,* the Chinese alligator *Alligator sinensis* (Wan et al. 2013), the American alligator *Alligator mississippiensis, *the gharial *Gavialis gangeticus* and the saltwater crocodile *Crocodylus porosus* (St John et al. 2012), the Burmese python *Python molurus* (Castoe et al. 2013), the King cobra *Ophiophagus hannah* (Vonk et al. 2013)*, *the speckled rattlesnake *Crotalus mitchellii* (Gilbert et al. 2014), and the corn snake *Pantherophis guttatus* (Ullate-Agote et al. 2015). According to the Genome 10k project, only nine additional reptilian genomes are in sequencing progress, still a low number to efficiently analyze the variety of developmental characters and evolutionary novelties encountered in this highly diverse group.

**Fig. 1.—:**
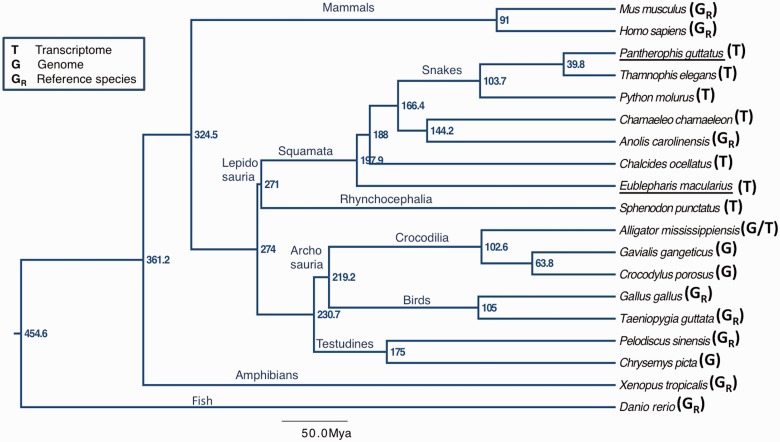
Chronogram among the selected reptilian and reference species used for annotation. The letters between parentheses after the species names indicate the data type (*T,* transcriptome; *G*, genome; *G_R_*, genome of a reference species). The underlined species were newly sequenced in our laboratory for this study. The tree topology and divergence times are based on the “TimeTree of Life” estimates (Hedges et al. 2006).

The aim of this study is to build the second version of the “Reptilian Transcriptomes Database” (http://www.reptilian-transcriptomes.org, last accessed June 10, 2015; Tzika et al. 2011), providing a high-quality and extensive annotation of transcriptomes and genomes from species that cover the major lineages of the Class Reptilia. We generated new transcriptomic data from two species, of interest for evolutionary developmental studies: The leopard gecko, *Eublepharis macularius*, and the corn snake, *Pa. guttatus*. For both species, Roche 454 and Illumina reads were sequenced from normalized cDNA libraries of multiple adult tissues (brain, kidneys, testes) and various embryonic stages. We also gathered published reptilian transcriptomic/genomic data sets, selecting representatives of the four extant orders (fig. 1): 1) Four Squamata, including two snakes (*P. molurus* [Castoe et al. 2011] and *Thamnophis elegans* [Schwartz et al. 2010]) and two lizards (*Chalcides ocellatus* [Brandley et al. 2012] and *Chamaeleo chamaeleon* [Bar-Yaacov et al. 2013]); 2) the single living tuatara species, *Sphenodon punctatus* (Miller et al. 2012); 3) three Crocodilia species (*Cr. porosus* [St John et al. 2012], *Al. mississippiensis* [Kunstner et al. 2010], and *G. gangeticus*); and 4) one Testudines (*Chr. picta* [Shaffer et al. 2013]).

Version 2 of the LANE runner software (Tzika et al. 2011) was implemented to annotate the selected and newly sequenced assemblies. LANE runner 2.0 integrates 1) iterative BLAST+ searches (Camacho et al. 2009) against multiple databases, 2) Reciprocal Best BLAST Hits (RBBH) identification for homology assessment, and 3) consensus sequence building to assemble sequences exhibiting the same annotation. This approach allowed us to annotate 50–70% of the transcripts from each data set, a higher percentage than in previously published transcriptomic studies. Using reference data sets, such as the Core Eukaryotic Genes Mapping Approach (CEGMA) (Parra et al. 2007), we show that combining transcriptomes of different tissues and developmental stages substantially increases the completeness of the assembled transcriptomes.

Using the annotated transcripts, we built concatenated protein alignments of single-copy families (up to 86,153 amino acids per species after quality trimming) and inferred maximum-likelihood phylogenetic trees that support the positions of turtles (Tzika et al. 2011) and the tuatara as sister groups of Archosauria and Squamata, respectively. The Reptilian Transcriptomes 2.0 resource is the largest transcriptomic database for reptiles to date and covers a large proportion of their diversity, including representatives of the four extant orders (Squamata, Rhynchocephalia, Crocodilia, and Testudines). This resource will be continuously updated to include selected new transcriptomic data as they become available, thus making it a reference for differential expression studies, comparative genomics/transcriptomics, linkage mapping, molecular ecology, and phylogenomic studies involving reptiles. The Reptilian Transcriptomes Database 2.0 is publicly available (www.reptilian-transcriptomes.org) for download and can be enquired through LANE runner 2.0 using a wwwblast server installed at the University of Geneva.

## Materials and Methods

### Transcriptomes Sequencing and Assembly

**Fig. 2.—:**
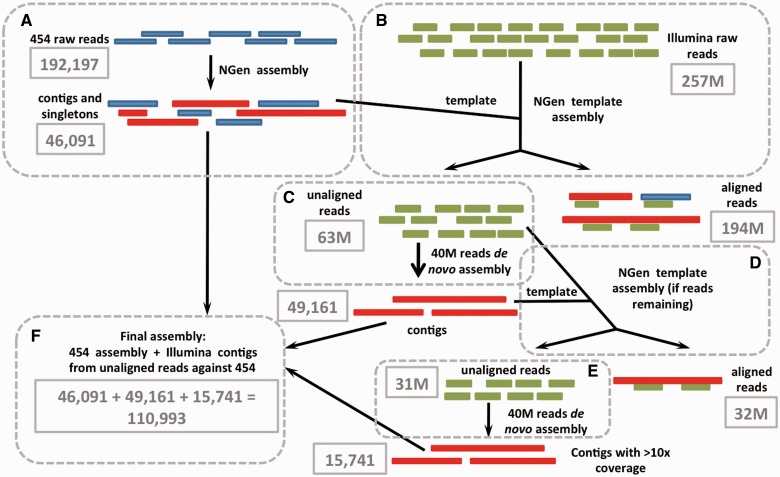
Assembly pipeline used to combine the “454” (in blue) and Illumina (in green) reads into nonredundant contigs (in red). Framed values correspond to those obtained for the *E. macularius* mix data set (multiple developmental stages and multiple adult organs), provided as an example. Dashed boxes delineate major steps of the assembly: (*A*) The “454” reads are de novo assembled, (*B*) the Illumina reads are aligned to the “454” assembly, and (*C–E*) iterative building of nonredundant Illumina contigs. The final assembly (*F*) includes both the “454” assembly and the Illumina contigs.

**Fig. 3.—:**
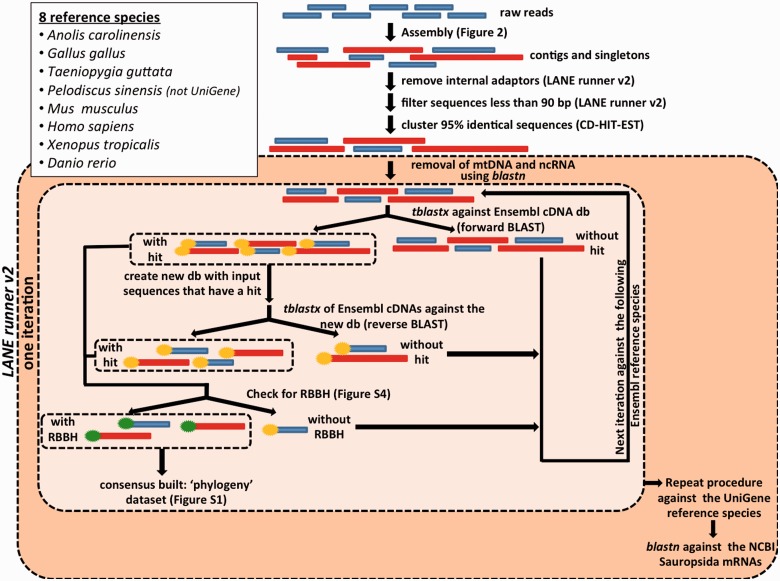
Outline of the transcriptome annotation pipeline. All steps included in the outer dash-framed box are performed in LANE runner. The steps of a single iteration (i.e., using one reference species) are grouped in the inner dashed frame. The reference species iteratively considered for annotation are listed in the inset. Query sequences having a hit are indicated with a yellow mark and those having an RBBH with a green mark.

We collected samples from individuals of our established *Pa. guttatus* and *E. macularius* colonies*.* Animal housing and samplings were performed in accordance with the Swiss animal welfare regulation (permit number 1008/3421/0). Normalized libraries (using the Trimmer-2 cDNA normalization kit; Evrogen) were prepared from 1) adult organs (testes, kidneys, and brain), and 2) embryos at two or three developmental stages (E10, E30, and E47 for *Pa. guttatus* and E8 and E24 for *E. macularius*). Each library was sequenced with the “454” (half-plate) and the Illumina (one lane, 100-base paired-end reads) technologies. For *Pa. guttatus*, we also included in the analyses our previously published vomeronasal organ (VNO) transcriptome (Brykczynska et al. 2013). We designed an assembly pipeline (incorporating SeqMan NGen v11.0; DNASTAR) presented in figure 2 to exclude redundancy among the “454” and Illumina contigs. Briefly, it comprises the following steps (detailed description in the supplementary methods, Supplementary Material online): 1) Assembly of the “454” reads (fig. 2*A*), 2) alignment of all the Illumina reads to the “454” assembly (fig. 2*B*), and 3) de novo and template assembly of the nonaligned Illumina reads (fig. 2*C*–*E*) in subsets of 40 million reads (due to computational resources limitation). The final assembly (fig. 2*F*) comprises the “454” assembly (contigs and singletons) and the Illumina contigs. Remaining adaptor sequences at the extremities or within contigs and singletons are removed with LANE runner. For each species, we assembled a mix of all cDNA libraries as well as each library (adults, embryos, or VNO) separately. To assess whether the mix assemblies are good representatives of the individual data sets, we compared the former with the latter by performing a template transcriptome assembly in NGen. Default parameters were used for template assemblies (with “other” as sequencing technology) except for the “Minimum Match Percentage” parameter that was set to 80. The mix assemblies were used for subsequent annotation. The “454” raw reads of the *Al. mississippiensis* brain transcriptome (Kunstner et al. 2010) were assembled with NGen, as well.

### Annotation of Transcriptomes

We annotated the transcriptomes and/or genomes of the following representatives of the four extant reptilian orders: 1) Six Squamata, including three snakes (*P. molurus, Pa. guttatus,* and *T. elegans*) and three lizards (*E. macularius, Cham. chamaeleon,* and *C. ocellatus*); 2) three Crocodilia (*G. gangeticus, Cr. porosus, *and *Al. mississippiensis*); 3) the single living Rhynchocephalia (*S. punctatus*); and 4) one Testudines (*Chr. picta*). Table 1 contains detailed information on the data sets and figure 1 shows the phylogenetic relationships among these species together with the reference species used for annotation (topology and divergence times based on the “Timetree of Life” estimates; Hedges et al. 2006). During our study, additional reptilian transcriptomes and genomes became available (e.g., Margres et al. 2013; Vonk et al. 2013; Wang et al. 2013); however, they either correspond to species closely related to the ones already selected or the transcriptomes originate from very specialized organs (e.g., the venom glands of snakes), in which case there is a high abundance of tissue-specific transcripts (e.g., coding for venom toxins) rather than a wide coverage of the species’ transcriptome. Similarly to the Reptilian Transcriptome 1.0 (Tzika et al. 2011), the annotation in the Reptilian Transcriptomes 2.0 is based on iterative BLAST searches, but with three major modifications: 1) The identification of RBBH to obtain a higher quality annotation (Altenhoff and Dessimoz 2009; Dalquen and Dessimoz 2013); 2) the use of the improved BLAST+ (release 2.2.28; Camacho et al. 2009), instead of the wwwblast web server; and 3) the use of Clustal Omega, instead of MUSCLE, as the multiple sequence alignment software for consensus building. The software LANE runner (Tzika et al. 2011) was upgraded (version 2.0) to accommodate these modifications. An overview of the annotation pipeline is presented in figure 3 and supplementary figure S1, Supplementary Material online, and a detailed description of the process is given in the supplementary methods and figure S2, Supplementary Material online.

**Table 1:** Reptilian Transcriptomes and Genomes Considered for Annotation

Species	Vernacular Name	Data Type	Source	Sequencing/Assembly	Size
*Chalcides ocellatus*	Ocellated skink	cDNA	Brandley et al. (2012)	Illumina assembly from uterus	300,966 contigs
*Sphenodon punctatus*	Tuatara	cDNA	Miller et al. (2012)	Illumina assembly from embryos	32,911 contigs
*Python molurus bivittatus*	Burmese python	cDNA	Castoe et al. (2011)	“454” assembly from liver and heart	37,245 contigs
*Chamaeleo chamaeleon*	Common chameleon	cDNA	Bar-Yaacov et al. (2013)	SOLiD assembly multitissue	164,692 contigs
*Thamnophis elegans*	Garter snake	cDNA	Schwartz et al. (2010)	“454” assembly multitissue and multi-individual	188,940 contigs and singletons
*Chrysemys picta*	Painted turtle	gDNA	Shaffer et al. (2013)	Genome assembly 3.0.1	6,080 scaffolds
*Gavialis gangeticus*	Gharial	gDNA	St John et al. (2012)	Draft genome assembly	47,351 scaffolds
*Alligator mississippiensis*	American alligator	gDNA	St John et al. (2012)	Draft genome assembly	8,897 scaffolds
*Crocodylus porosus*	Saltwater crocodile	gDNA	St John et al. (2012)	Draft genome assembly	23,365 scaffolds
*Alligator mississippiensis*	American alligator	cDNA	Kunstner et al. (2010)	Brain “454” reads	438,029 reads
*Pantherophis guttatus*	Corn snake	cDNA	Brykczynska et al. (2013)	VNO “454” and Illumina libraries	343,062 and 54.4M reads from “454” and Illumina
*Pantherophis guttatus*	Corn snake	cDNA	This study	Adults testes, brain and kidneys “454” and Illumina paired-end libraries	135,630 and 145M reads from “454” and Illumina
*Pantherophis guttatus*	Corn snake	cDNA	This study	Embryonic “454” and Illumina paired-end libraries	45,417 and 129.8M reads from “454” and Illumina
*Eublepharis macularius*	Leopard gecko	cDNA	This study	Adults testes, brain and kidneys “454” and Illumina paired-end libraries	112,760 and 128M reads from “454” and Illumina
*Eublepharis macularius*	Leopard gecko	cDNA	This study	Embryonic “454” and Illumina paired-end libraries	79,437 and 129.8M reads from “454” and Illumina

### Consensus Sequences

Consensus sequences were built using LANE runner 2.0 as follows: 1) The best match of each input sequence is identified considering the ordered criteria of *e*-value, match length, and % identity; 2) input sequences matching with the same database sequence are aligned using Clustal Omega (Sievers and Higgins 2014); 3) a majority consensus is built, where the database sequence is used only as an anchoring point to define the relative positions of the input sequences; and 4) the consensus is named after the database sequence. When several input sequences match with a single database sequence, we obtain a “one-to-many consensus.” When a single input sequence matches a database sequence, it is labeled as a “one-to-one” consensus, where no alignment is necessary and the input sequence takes the name of the database sequence. Contrary to LANE runner v1 (Tzika et al. 2011) that used MUSCLE (Edgar 2004), LANE runner 2.0 uses Clustal Omega which is better adapted to the alignment of small fragments against a long reference sequence.

Two data sets were generated and used to build consensus sequences (fig. 3 and supplementary fig. S1, Supplementary Material online). The “phylogeny data set” consisted of the RBBH against the Ensembl cDNA and the UniGene databases. The “annotation data set” combined 1) the phylogeny data set, 2) the “NCBI Sauropsida mRNAs” BLASTn results, and 3) the non-RBBH Ensembl and UniGene database matches. The phylogeny data set corresponds to a high-quality annotation that can be used to build phylogenetic trees, whereas the annotation data set maximizes the number of annotated transcripts in the species investigated.

### Genome Annotation

We developed a simple method to partially annotate the following genomes for which annotation is not yet available in the literature or public databases: *Al. mississippiensis, G. gangeticus, Cr. porosus* (St John et al. 2012), and *Chr. picta* (Shaffer et al. 2013). The pipeline is outlined in supplementary figure S3, Supplementary Material online, with *Chrysemys* as an example. First, we used BioMart (Smedley et al. 2009) to extract the exons, as well as the 5′ and 3′ 1,000-base flanks of *Pelodiscus* Ensembl cDNAs. Then, we performed an NGen assembly where the *Chrysemys* genome was used as template and the *Pelodiscus* exons and flank sequences as reads (default NGen parameters, “Other” as sequencing technology, and Minimum Match Percentage set to 80). Using the “mpileup” option of samtools (Li et al. 2009), we extracted the genome segments that align to the *Pelodiscus* exons and flanks. These sequences are potential exons (including 3′- and 5′- untranslated regions [UTRs]) of the *Chrysemys* genome that we want to annotate. To assemble the potential exons into transcripts, a BLASTn search was performed against the *Pelodiscus* cDNA database. The BLASTn search settings were the same as for the mtDNA comparison (see supplementary methods, Supplementary Material online), without considering a minimum match length threshold to permit the alignment of even the shortest potential exons. As in our annotations of transcriptomes, consensus sequences were built for the exons that had a hit against the cDNA database (supplementary fig. S3, Supplementary Material online). The same procedure was used for annotation of the genomes of the three crocodilian species except that both *Gallus *and *Pelodiscus* exons were aligned to each crocodilian genome, and both *Gallus* and *Pelodiscus* cDNA databases were used in the BLASTn search. Annotated genes were restricted to a phylogeny data set by keeping only: 1) Consensus sequences named after a *Gallus *transcript and greater than 90 bp for the crocodilian species, and 2) consensus sequences greater than 90 bp for *Chr. picta*.

### Annotation of the *Pa. guttatus* and *E. macularius* Individual Libraries

For *Pa. guttatus* and *E. macularius*, we separately sequenced cDNA libraries from different organs and embryonic developmental stages. For each of the two species, all reads from all libraries were pooled before performing assembly and annotation (figs. 2 and 3). We then investigated, with a tBLASTx search (same settings as for the Ensembl cDNA searches), the contribution of the individual libraries to the annotation data set and to the “orphans” generated with the mixed annotation.

## Results

### Assembly of the Corn Snake, Leopard Gecko, and American Alligator Transcriptomes

For *Pa. guttatus* and *E. macularius*, we used the “454” and Illumina technologies to sequence a multitissue normalized library (kidney, testes, and brain) from three adult individuals, as well as one normalized library from embryos of various developmental stages. In addition, the VNO reads that we sequenced for *Pa. guttatus* (Brykczynska et al. 2013) were included in the analysis. The number of “454” reads obtained for each library varies between 45,000 and 343,000 (table 2), with an average read length of 219–401 bp. The number of Illumina 100-base paired-end reads ranged from 128 millions (M) in *E. macularius* (adult data set) to 145M in *Pa. guttatus* (adult). The individual (adults, embryos, VNO) and mixed (all reads) data sets obtained with the “454” technology were assembled using NGen (fig. 2*A*). The percentage of reads discarded due to quality filtering and trimming varied from 0.6% to 25.8% (table 2). The same approach was used to assemble the *Al. mississippiensis* brain transcriptome “454” reads (Kunstner et al. 2010; SRR029332). The “454” assembly was then used as template to remove redundant Illumina reads (fig. 2*B*). Depending on the data set, 48–75% Illumina reads matched with the “454” assembly, this percentage being higher for the *E. macularius* than for the *Pa. guttatus* assemblies. Forty millions of the remaining unaligned Illumina reads were then de novo assembled (fig. 2*C*). This assembly was used as a template for all unaligned Illumina reads (fig. 2*D*). Yet unassembled Illumina reads were de novo assembled by batches of 40M (fig. 2*E*). The final assemblies consisted of the “454” contigs and singletons and the Illumina contigs. LANE runner 2.0 was used to remove adaptor sequences within the contigs. More than 95% of contigs/singletons of the individual data sets aligned to the mix assembly sequence (supplementary table S2, Supplementary Material online), thus the latter was used for subsequent annotation.

**Table 2:** NGen Assembly Workflow Statistics

	*Pantherophis guttatus*				*Eublepharis macularius*		
	Adults	Embryos	VNO	Mix	Adults	Embryos	Mix
Number of plates	1	1	1	3	1	1	2
454 reads	135,630	45,417	343,062	524,109	112,760	79,437	192,197
454 discarded	8,557 (6.3%)	4,374 (9.6%)	1,912 (0.6%)	15,071 (2.9%)	29,056 (25.8%)	4,466 (5.6%)	33,522 (17.4%)
454 contigs	17,570	6,133	38,666	45,955	10,635	6,595	17,876
454 singletons	27,826	17,069	56,632	98,265	26,200	13,434	28,215
Av. contig length	556	499	447	497	393	545	712
Greater than 500 bp	9,585	2,951	12,369	17,075	3,192	3,439	13,122
Greater than 1 Kb	1,471	343	1,325	3,667	265	595	3,404
454 assembly	45,396	23,202	95,298	144,220	36,835	20,029	46,091
Number of lanes	0.5	0.5	0.5	1.5	0.5	0.5	1
Illumina reads	145M	129.8M	54.4M	329.2M	128M	129.8M	257.8M
Aligned to 454	92M (63%)	62.8M (48%)	32.3M (59%)	216.9M (66%)	82M (64%)	94.8M (73%)	194.2M (75%)
Illumina contigs	63,227	86,371	28,453	134,457	50,737	36,254	64,902
Av. contig length	423	394	575	396	410	454	389
Greater than 500 bp	20,805	25,892	14,891	39,300	15,131	13,668	18,577
Greater than 1 Kb	2,738	3,570	2,652	3,589	953	2,097	1,831
Number iterations	1	2	1	3	1	1	2
Final assembly	108,623	109,573	123,751	278,677	87,572	56,283	110,993
After adaptor removal	108,678	109,589	124,012	279,699	87,703	56,302	111,237

Note.—The two first shaded rows correspond to the total number of contigs/singletons obtained from "454" and Illumina reads, and the third shaded row corresponds to the total number of contig/singletons after removal of adaptors.

### Annotation of the Transcriptomes

Along with these three newly assembled reptilian transcriptomes (corn snake, leopard gecko, and American alligator), another five transcriptomes, originating from different tissues and developmental stages, were annotated: *Cham. chamaeleon*, *T. elegans*, *P. molurus*, *S. punctatus*, and *C. ocellatus* (fig. 1 and table 1). First, we preprocessed the assemblies by 1) removing sequences of ≤ 90 nucleotides length and 2) clustering ≥ 95% identical sequences using CD-HIT-EST (Li and Godzik 2006; Fu et al. 2012) by maintaining only the longest sequence from each cluster (fig. 3).

**Fig. 4.—:**
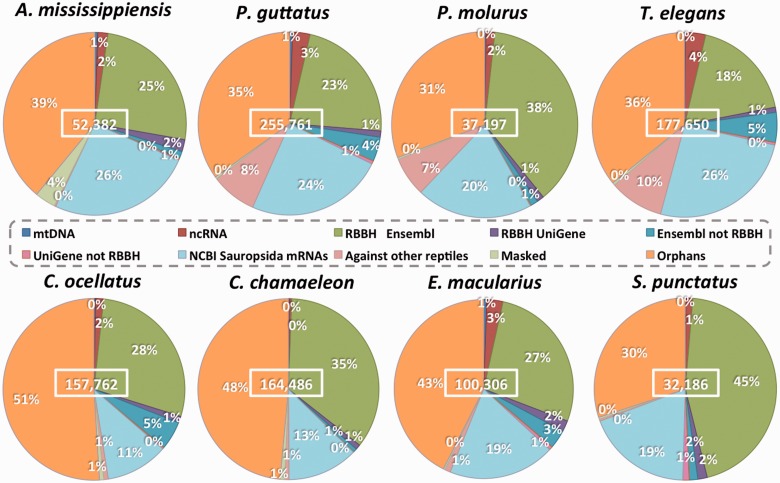
Piecharts showing the percentage of contig/singletons annotated at each step of the pipeline. The number of input sequences for each transcriptome is indicated in the middle of each graph.

Our annotation pipeline (fig. 3 and supplementary fig. S1, Supplementary Material online) consists of iterative BLAST searches and identification of RBBH for a higher quality result, all steps being performed with the updated version of LANE runner (version 2.0). The filtered contigs and singletons of each reptilian transcriptome were compared with a BLASTn search to 1) the mitochondrial genome of the corresponding species or that of a closely related one and 2) a database including Ensembl v73 ncRNA sequences from eight reference species: *A. carolinensis*, *Gallus gallus*, *Taeniopygia guttata*, *Pe. sinensis, Mus musculus*, *Homo sapiens*, *Xenopus tropicalis,* and *Danio rerio* (hereafter named *Anolis, Gallus, Taeniopygia, Pelodiscus Mus, Homo, Xenopus* and *Danio*, to distinguish them as reference species from the annotated ones)*. *Less than 1% of contigs and singletons corresponded to mtDNA sequences and only 1–4% to ncRNA (fig. 4). RBBH were identified with tBLASTx searches against the Ensembl v73 coding cDNA and “UniGene November 2013” databases of each reference species (fig. 3; see Supplementary Material online for details). Second, the sequences still nonannotated by this iterative process were aligned with tBLASTx against an “NCBI (National Center for Biotechnology Information) November 2013” database containing mRNA sequences from Sauropsida. The annotated sequences were divided into two data sets: 1) An annotation data set comprising all annotated sequences (with and without RBBH) and 2) a high quality phylogeny data set including only the annotated sequences with a RBBH. In a last round of BLAST searches, the nonannotated contigs/singletons of each reptilian species were compared with the annotation data set and the nonannotated sequences of the other seven reptilian transcriptomes. Finally, the remaining sequences were masked using RepeatMasker to check whether their lack of annotation was due to the presence of repetitive elements. The sequences lacking annotation after all these processes are called orphans (supplementary fig. S1, Supplementary Material online).

As shown in figure 4, the annotation success is high but varies among species, with the number of orphans ranging from 30% in *S. punctatus* to 51% in *C. ocellatus*. Compared with the Reptilian Transcriptome v1, this percentage is improved (i.e., reduced), in part due to the BLAST search step against the NCBI Sauropsida mRNAs, which include transcripts of reptilian species closely related to those under study here. This step indeed resulted in the annotation of 11 to 26% of the filtered contigs and singletons, the percentage being highest for the transcriptomes of snakes and *Al. mississippiensis*. On the other hand, the UniGene database provided little improvement in the annotation (1 to 3% of the total), probably because of its high overlap with the Ensembl cDNA database. The percentage of input sequences with a RBBH varies from 19 to 47%, with the greatest proportions found in *S. punctatus* and *P. molurus* transcriptomes.

**Fig. 5.—:**
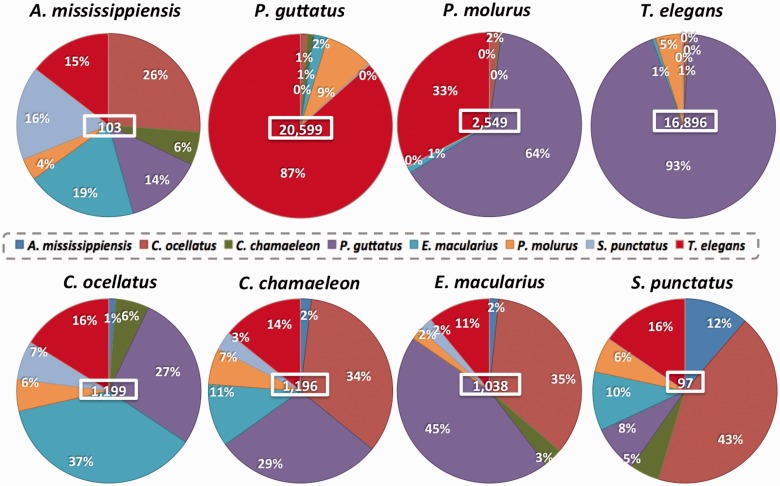
Piecharts showing the percentage of nonannotated contigs/singletons that match with the other annotated reptilian transcriptomes. The total number of hits is indicated in the middle of each graph.

The BLAST searches among the reptilian transcriptomes substantially improved the snakes’ annotation (figs. 4 and 5), as 7 to 10% of their contigs/singletons matched with other transcriptomes, mainly those of other snakes. In particular, 93% of the *T. elegans* sequences still nonannotated after BLAST searches against Ensembl, UniGene and NCBI Sauropsida mRNAs, matched a *Pa. guttatus* sequence (fig. 5). Similar results are obtained for the reverse comparison (87% of the nonannotated *Pa. guttatus* sequences matched *T. elegans*). These results strongly suggest that these sequences represent snake-specific transcripts. For the other transcriptomes, only a small number (<1,200) of contigs/singletons were annotated, probably because none of these reptiles is more closely related to each others than to one of the Archosauria or Squamata reference species (*Anolis* and *Gallus*) (fig. 1). Finally, very few repetitive elements were identified, with the highest percentage of masked sequences in *Al. mississippiensis* (4%) and ≤1% for all the other species.

Even though the number of input sequences annotated here from each reptilian species is greater compared with the Reptilian Transcriptome v1 and other transcriptomic studies, the percentage of orphans is still substantial, which can be due to a combination of factors such as 1) the absence of full genome sequence data from the particular species for extensive annotation, 2) the presence of sequencing or assembly artifacts, 3) the presence of genomic contaminations in cDNA libraries, 4) the large evolutionary distance of the eight reptilian species from the reference species (fig. 1)—*Anolis* being the most closely related reference species to Squamata (144–197 Myr) and to *S. punctatus* (271 Ma), whereas *Gallus *is the most closely related to *Al. mississippiensis* (219 Myr)—and 5) the presence of real orphans (also known as taxonomically restricted genes), which lack sequence similarity or even homology to other species’ genes and, depending on the organism, may correspond to 10–20% of all genes (Khalturin et al. 2009; Tautz and Domazet-Loso 2011).

In an attempt to rule out some of these hypotheses, we compared the orphans with available reptilian genomes. We mapped all but 87 contigs of the 11,435 *P. molurus* orphans, 98.1% of the 89,329 *Pa. guttatus* orphans, and 95% of the 20,441 *Al. mississippiensis* orphans to their respective genomes using NGen. We obtained similar results when performing a BLASTn search against the genome of each corresponding species. Thus, at least for these three species, the vast majority of nonannotated contigs/singletons are not artifacts but must be either real transcripts or genomic DNA contaminations (introns, intergenic regions). Furthermore, we aligned the *Pa. guttatus* and *T. elegans* orphans to the two snake genomes available: *P. molurus* (100 Myr) and the king cobra *O. hannah* (44 Myr). Twenty-six percent out of 63,631 *T. elegans* orphans and 30% out of 89,329 *Pa. guttatus* orphans were aligned to the *P. molurus* genome, whereas these percentages greatly increased (68% and 74%) for the comparison with the more closely related *O. hannah* genome. These results provide further support that these orphans are real transcripts.

Finally, we compared the quality statistics of the annotated contigs/singletons and the orphans (supplementary table S3, Supplementary Material online) with a two-sample *t*-test. In all transcriptomes, the average length of the annotated sequences is significantly greater than that of the orphans (*P* value < 0.001). This measure remains significant even if we consider separately the average length of contigs and singletons. The contig coverage (average number of reads per contig) is also significantly higher for the annotated than for the orphan contigs (*P* value < 0.001). For example, in *P. molurus* and *T. elegans*, there is almost a 5-fold coverage difference between annotated contigs and orphans.

### Consensus Sequences Statistics

Following the BLAST searches, we used LANE runner 2.0 to assemble “consensuses” (see Materials and Methods for details). When a single input sequence matches a database sequence, it is labeled as a “one-to-one consensus” and the input sequence takes the name of the database sequence. When multiple contigs/singletons match the same database sequence, LANE runner 2.0 performs Clustal Omega (Sievers and Higgins 2014) alignments to define the relative positions of the input sequences and assembles them into a “one-to-many consensus.”

Overall, 20–39% of the consensuses are “one-to-many” (supplementary table S4, Supplementary Material online) with an average transcript coverage of 18–40%. In the case of the Ensembl phylogeny data set, we observe a greater percentage of “one-to-many consensuses” (28–54%) and a large mean coverage of these transcripts (19–39%). To investigate whether the coverage of transcripts in all our analyzed data sets is 3′- or 5′-biased, we split the “one-to-many consensuses” against *Anolis* genes into three parts: The 3′ 30%, the middle 40%, and the 5′ 30%. We observed that in the 3′- and 5′-ends, the percentage of gaps ranged from 64% to 90% versus 49–72% in the middle. As we extended the *Anolis* cDNAs with 1,000-bases upstream and downstream of the CDS (in order to include the missing UTRs), the lower coverage of these regions suggests that some of them are not part of the real mRNAs or are highly variable between distantly related species.

**Fig. 6.—:**
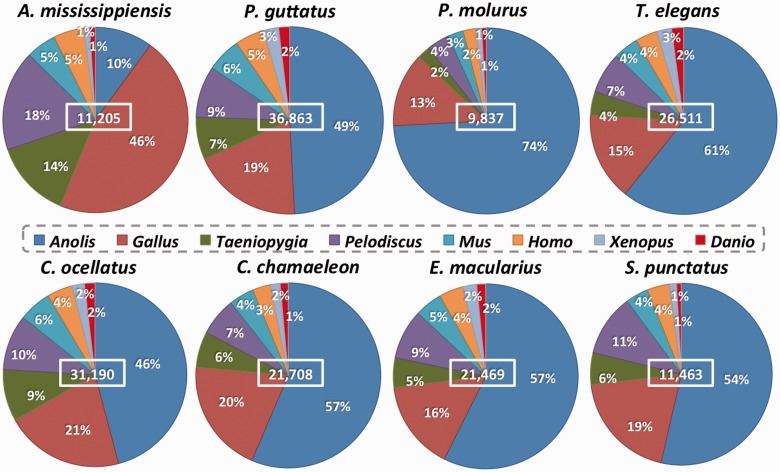
Piecharts showing the percentage of consensus sequences annotated with each reference species in the Ensembl or UniGene databases. The total number of consensuses is indicated in the middle of each graph.

In figure 6, we show the distribution of the consensus sequences named after an Ensembl or UniGene reference species gene. For all transcriptomes, most consensus sequences are named after the first reference species (used in the iterative BLAST searches) selected to be the most closely related to the species being annotated: *Gallus* is used for the annotation of 46% of the *Al. mississippiensis* sequences and *Anolis* is used for 46–74% of the sequences for the other reptilian species. For all the data sets, the combination of *Anolis* and *Gallus* sequences used as reference to build consensuses ranged from 56% to 87%. In all cases, there are more consensuses named after a *Pelodiscus* cDNA than after a *Taeniopygia* one, underlying the importance of using reference species from different taxonomic groups rather than several species from the same one. The remaining four non-Sauropsida species were used as reference to build only about 10% of the consensus sequences, due to their greater evolutionary distance.

### Annotation of Genomes

We additionally annotated the genomes of three Crocodilia (*Al. mississippiensis*, *Cr. porosus,* and *G. gangeticus*) and a turtle (*Chr. picta*) using *Gallus* and *Pelodiscus* exons extracted from Ensembl (supplementary fig. S3, Supplementary Material online). We selected *Chr. picta* because there is a preliminary gene annotation in “Ensembl Pre!” that can help us assess the efficiency and quality of our annotation pipeline. *Pelodiscus* exons and flanks, as well as *Gallus *exons were used to identify the corresponding Crocodilia exons, whereas only the *Pelodiscus* sequences were used for the *Chr. picta* genome annotation. Seventy percent of *Pelodiscus* sequences were aligned to the *Chr. picta* genome, resulting in 143,467 potential *Chr. picta* exons (supplementary table S5, Supplementary Material online). Almost all (99.8%) were assembled to transcripts after a BLASTn search against the Ensembl *Pelodiscus* coding cDNA database, resulting in 18,447 consensus sequences. To assess the quality of this annotation, the consensuses were compared with the *Chr. picta* preliminary gene annotation of Ensembl: 15,028 of 18,447 (81.3%) consensus sequences had a BLASTn hit (using the same settings as for the identification of mtDNA transcripts, but increasing the minimum %ID to 90% and lowering the *e*-value threshold to 10^−^^5^ to be more stringent), demonstrating the efficiency of our annotation pipeline.

Only 33% of the *Pelodiscus* and *Gallus *exons were aligned to the Crocodilia genomes (supplementary table S5, Supplementary Material online), probably because none of the reference species is closely related to these crocodilian genomes and, therefore, only well-conserved genes can be identified. For each Crocodilia, around 90,000 potential exons were retrieved and almost all of them (99.7–99.8) were assembled against a *Gallus *or a *Pelodiscus* coding cDNA after a BLASTn search, yielding more than 17,000 consensus sequences. Note that the percentage of “one-to-many consensuses” (67% for Crocodilia and 87% for *Chr. picta*) in these annotations of genomes is higher than in the annotations of transcriptomes. For the phylogeny data set (built only with sequences greater than 90 bp and, for Crocodilia, only with sequences named after a *Gallus *cDNA), the percentage of “one-to-many consensuses” is even higher (around 87% in the Crocodilia and 91% in *Chr. picta*), indicating that most of the ≤90 bp short sequences correspond to “one-to-one consensuses.” Furthermore, there are 11,709 consensuses named after the same database sequence in *Al. mississippiensis*, *G. gangeticus* and *Cr. porosus*, possibly corresponding to well-conserved transcripts at this taxonomic level.

### Comparison with Reference Data Sets

**Fig. 7.—:**
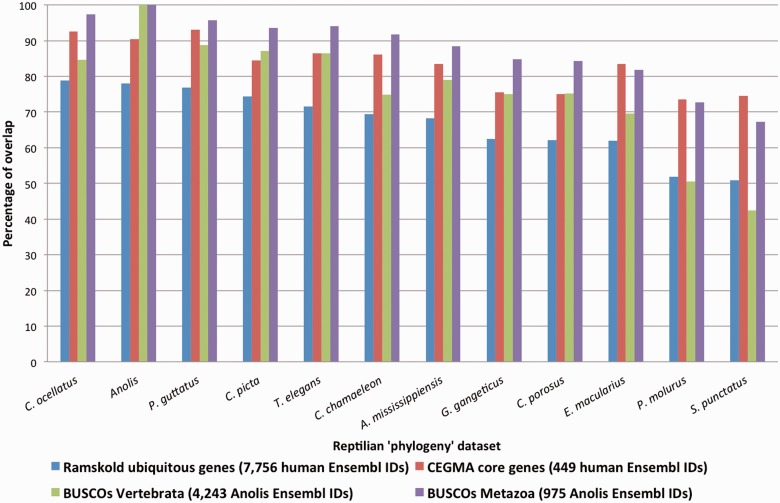
Completeness of the annotated transcriptomes assessed with four reference data sets: Ramskold ubiquitously expressed genes in human (blue bars), CEGMA core human genes (red bars), OrthoDB7 BUSCOs from the vertebrate (green bars), and the metazoan (purple bars) radiation nodes. The species are ordered from higher to lower overlap with the Ramskold data set and *Anolis* is shown as reference.

We verified the quality of our annotations by assessing their completeness against four established data sets (fig. 7): A set of ubiquitously expressed genes in human tissues and cell lines (Ramskold et al. 2009), a selection of eukaryotic genes from the CEGMA (Parra et al. 2007), and two “Benchmarking sets of Universal Single-Copy Orthologs” (BUSCOs; from OrthoDB7 [Waterhouse et al. 2013]) in Vertebrata and Metazoa. The comparisons were performed by BLASTn searches of the reference data sets against the consensuses of the phylogeny data sets. In the case of *Al. mississippiensis*, the results of the transcriptome and genome annotations were combined.

Ramskold et al. (2009) identified 7,756 human Ensembl genes ubiquitously expressed in 16 organs and cell lines. These are mainly intracellular housekeeping genes involved in basic functions, such as metabolism, transcription or macromolecule synthesis. We identified roughly 80% of these genes in the *Anolis* reference genome. Note that it is unknown whether all of these genes are ubiquitously expressed in the corresponding 16 *Anolis* tissues/organs. We show the overlap with the Ramskold et al.’s core set of genes to be slightly higher for the *C. ocellatus* transcriptome annotation than for the *Anolis* genome (fig. 7), underlying the quality of our *C. ocellatus* annotation. On the other hand, *S. punctatus* and *P. molurus* presented a substantially lower percentage (about 50%) of correspondence with this Ramskold et al.’s core set of genes. For all other species investigated, the percentages of overlap were intermediate (62–77%). The best percentage among Crocodilia was found for *Al. mississippiensis* (68%), possibly because transcriptomic and genomic data were combined for this species.

The CEGMA data set includes a set of 456 human transcripts that are highly conserved in a wide range of eukaryotic taxa and has been previously used to assess the quality of genome annotations (Parra et al. 2007). CEGMA was built considering conserved genes in six organisms: *Homo sapiens, Drosophila melanogaster*, *Arabidopsis thaliana*, *Caenorhabditis elegans*, *Saccharomyces cerevisiae* and *Schizosaccharomyces pombe*, 449 of which have an Ensembl human identifier. Despite this high conservation among taxa, we identified only 90% of these genes in the *Anolis* reference genome (fig. 7). The high percentage (>92%) of these genes identified in *Pa. guttatus* and *C. ocellatus* indicates the high-quality annotation we achieved here. The other species exhibited an overlap with the CEGMA data set between 73% and 86% (the lowest being, again, for *S. puctatus* and *P. molurus*).

Finally, the two BUSCOs data sets, provided by OrthoDB7 (Waterhouse et al. 2013) for the Metazoan and Vertebrata radiation nodes, correspond to sequences from orthologous groups with single-copy genes present in more than 90% of the species of the corresponding radiation node. There are 41 species in the Vertebrata BUSCOs (including *Gallus *and *Anolis*) and 93 species in the Metazoan BUSCOs data sets (with 4,243 and 975 *Anolis* Ensembl identifiers, respectively). The Metazoan BUSCOs are a subset of the Vertebrate data set and correspond to genes conserved over a greater evolutionary period, explaining that we identify a substantially greater percentage of genes in the Metazoan (67–97%; purple columns, fig. 7) than in the vertebrate (42–89%; green columns) BUSCOs.

In short, and as supported by all reference data sets (fig. 7), *Pa. guttatus*, *T. elegans*, *C. ocellatus* and *Chr. picta* data sets have the most complete annotations, whereas *S. punctatus* and *P. molurus* exhibit substantially less complete annotations. These results are consistent with the source of the corresponding transcriptomes: The *S. punctatus* transcriptome is from a nonnormalized library of a single early stage embryo (at which developmental point a restricted panel of genes is probably expressed) and the *P. molurus* transcriptome is from nonnormalized libraries of heart and liver, two organs in which a high proportion of mRNAs correspond to a low number of different transcripts (Ramskold et al. 2009). The *Pa. guttatus* and *E. macularius* transcriptomes, newly sequenced for this study, also exhibit substantial differences in their annotation completeness. The *Pa. guttatus* transcriptome is more complete for two reasons: 1) The availability of the VNO transcriptome of *Pa. guttatus* doubles the number of contigs/singletons in that species, and 2) using three embryonic stages in *Pa. guttatus*, instead of two in *E. macularius*, probably enriched the transcript pool further. Note that, in general, the full length of the reference sequences was partially covered by the aligned reptilian transcripts: CEGMA mean coverage: 29–65%, Ramskold: 25–64%, BUSCOs Vertebrata: 28–69%, and BUSCOs Metazoa: 33–75%.

### Large Phylogeny Inference

The phylogenetic positions of the tuatara *S. punctatus* and Testudines (turtles) have long been debated. The first phylogenomic analysis to investigate the question of the position of turtles (Tzika et al. 2011) supported Testudines as the sister group to Archosauria. Two subsequent phylogenomic analyses, based on nucleotide/amino-acid (Chiari et al. 2012) or ultraconserved-element alignments (Crawford et al. 2012), confirmed this hypothesis. Here, using the Reptilian Transcriptomes 2.0, consisting of a greater number of species than the first version and of better annotated transcripts, we built yet improved data sets (up to 86,153 amino acids per species after quality trimming) and inferred maximum-likelihood phylogenomic trees that support the positions of turtles (Tzika et al. 2011) and the tuatara as sister groups of Archosauria and Squamata, respectively (supplementary results, supplementary methods, table S1, and figs. S4 and S5, Supplementary Material online).

## Discussion

Here, we present a pipeline for the annotation of transcriptomic and genomic data sets, which we applied to build the second version of the Reptilian Transcriptomes Database. To that end, the LANE runner software was updated with the incorporation of new functionalities that allowed us to 1) perform iterative BLAST searches using the BLAST+ program against multiple databases and reference species covering a wide evolutionary range within vertebrates, 2) identify RBBH, and 3) build consensus sequences by joining the contigs/singletons that correspond to the same reference sequence. This approach fulfilled our three objectives: 1) The production of a high-quality annotation, thanks to the identification of RBBHs, which can be used for phylogenomic analyses (phylogeny data set); 2) the generation of an exhaustive annotation resource (annotation data set); and 3) the use of a single workflow for the analysis of all data sets, independently of the species, tissues, or sequencing technology, greatly facilitating comparative analyses. This new annotation resource is the largest transcriptomic database for reptiles to date and covers a large proportion of their diversity, including representatives of the four extant orders (Squamata, Rhynchocephalia, Crocodilia, and Testudines). Furthermore, its structure facilitates the incorporation of new data sets as they are produced, making it an expansible resource rather than a static list of genes. The Reptilian Transcriptomes Database 2.0 is available at www.reptilian-transcriptomes.org.

Using our transcriptome annotation pipeline, we annotated between 50% and 70% of the nonredundant contigs and singletons for each species. For the reannotated transcriptomes, this percentage is in all cases higher than in the corresponding original publication, underlying the efficiency of our approach. For instance, Schwartz et al. (2010) annotated 25% of the garter snake *T. elegans* transcripts, whereas, using our workflow, we increased this value to 64%. Note, however, that the only reptilian reference at the time of that original publication was the draft genome of *Anolis*. But even in the case of the *Cham. chamaeleon* transcriptome, that was recently published, we doubled the number of annotated transcripts (from 25% to 50%) (Bar-Yaacov et al. 2013). For the newly sequenced *Pa. guttatus* transcriptome presented here, not only does it contain more contigs/singletons than the first version (79,688 transcripts in version 1 vs. 255,761 transcripts in version 2), but it also annotates a greater percentage of them (46% in the first vs. 65% in the second version). The better performances of our updated pipeline are due to 1) the addition of Illumina data, increasing the coverage of each transcript; 2) the sequencing of transcripts from multiple adult tissues and embryonic stages; and 3) an improvement in the annotation thanks to the use of new reference databases.

Even though we obtained a higher percentage of annotation than in previous studies, there is still a substantial proportion of orphan contigs. Our comparisons with the corresponding species genome or with genomes of closely related species suggest that these orphans are unlikely to be sequencing or assembly errors, as most show a hit to a genomic region. Therefore, there are two explanations for the lack of annotation of these sequences. First, some of these sequences may represent highly variable regions of transcripts (hence, difficult to annotate with similarity searches), either because they are not translated (UTRs) or because they do not code for functionally constrained protein domains. Second, many of these sequences are likely to be real orphans, that is, taxonomically restricted genes, which emerged recently in time, hence, lack homology to divergent species genes (Khalturin et al. 2009; Tautz and Domazet-Loso 2011). It was long assumed that such orphan genes appear due to duplication events followed by rapid divergence, but a recent study proposed an alternative mechanism of de novo emergence from noncoding DNA regions (Neme and Tautz 2013), favoring the appearance of short genes and open-reading frames. In addition, comparisons among the transcriptomes from different species of snakes in our database confirmed that a substantial proportion of orphans are taxonomically restricted genes in that lineage as 7–10% could be identified through similarity searches among snake transcriptomes. These pools of taxonomically restricted genes are therefore of particular interest because they may be involved in lineage-specific adaptations. However, until high-quality genomic data from more closely related species are available, the identification of taxonomically restricted genes remains a challenge.

Our genome annotation pipeline proved to be a simple and accurate method to identify a large proportion of genuine transcripts. Even though it is not as exhaustive as available genome prediction pipelines, which combine genome sequencing and RNA-Seq data from the same species to identify genes, our database and corresponding pipeline provide a first good draft annotation before full genome sequencing data becomes available. The high overlap (81.3%) between the *Chr. picta* cDNA set from Ensembl Pre! and our data set proves the efficiency of our method, especially when the annotation of closely related species is available.

We also observed a high variability, among species, in the completeness of the annotation. In the case of genome annotation, the well-conserved transcripts were more easily retrieved than the fast evolving ones. Regarding the transcriptome annotation, the variability in the quality of the results is associated with the specific approach used to generate the raw data: These transcriptomes were obtained from different tissues and using different sequencing and assembly techniques. Comparisons against reference data sets (CEGMA core genes, BUSCOs, and ubiquitously expressed genes) showed that the *Pa. guttatus* and *C. ocellatus* transcriptomes are the most complete, whereas the ones of *S. punctatus* and *P. molurus* present the lowest annotation and coverage. Moreover, even though we identified numerous transcripts (on average 35,912 per species), they cover a variable proportion (per-species average of 18–54%) of the full-length cDNAs.

Several modifications of our pipeline could yet increase the speed and quality of annotation. We could optimize the selection of reference databases as, for example, there is a great overlap between the Ensembl and UniGene databases, making comparisons with the second one highly redundant. Even more, the reliability and possible function of orphans could be analyzed by identifying open-reading frames and protein motifs/domains using databases, such as InterPro, SMART, Pfam, PANTHER, and PROSITE.

Using our transcriptome annotation, we built very large alignments of concatenated homologous protein sequences (from single-copy gene families) and generated the best- supported phylogeny to date among the five major lineages of Sauropsida. All analyses strongly support the position of turtles as the sister group of Archosauria (Tzika et al. 2011) and the position of the tuatara as the sister group of Squamata (both, long-standing questions).

In conclusion, the Reptilian Transcriptomes Database 2.0 presented here is a useful resource for comparative genomics/transcriptomics, differential expression analyses, as well as molecular ecology, developmental and evolutionary studies involving reptiles. For instance, this database can be used as a reference for RNA-Seq and in situ hybridization studies aiming to identify genes that are differentially expressed in distinct cell types, tissues or phenotypes. Such studies are not possible in the absence of a high-quality annotated transcriptome/genome from either the species of interest or a closely-related one.

As additional data accumulate, we will expand and improve the Reptilian-Transcriptomes.org resource. Additional transcriptomes of other reptilian species will undoubtedly become available. The Order Squamata, which comprises 9,372 species (i.e., 95% of all reptiles), would particularly warrant further sequencing efforts. We will also add new reptilian reference species in our pipeline as the annotation of their genomes becomes available, and we will continue updating the database by 1) annotating and including new transcriptomes and 2) improving the quality and completeness of the ones that are already present in the database. Finally, as sequencing throughput and quality will continue to improve, we eventually aim at building a reptilian “phylome” (Huerta-Cepas et al. 2007) that could be incorporated into MANTiS, a software that allows users to explore and interrogate genome content and associated functional data within a phylogenetic context (Tzika et al. 2008).

## Supplementary Material

Supplementary methods, results, figures S1–S5, tables S1–S5, and bibliography are available at *Genome Biology and Evolution* online (http://www.gbe.oxfordjournals.org/).
